# Supplementary material for: Tead1a Initiates Transcriptional Priming Through the TEAD1a/YAP‐Notch1‐Spi1/Cebpα Axis to Promote Neutrophil Fate
Source: Adv Sci (Weinh). 2025 Aug 28;12(41):e05441. doi: 10.1002/advs.202505441 (PMC12591127; doi:10.1002/advs.202505441)
Supplement: Supplementary file 4 — Supplementary Dataset [file ADVS-12-e05441-s004.zip › 3-3.zip!/3-3/02-3.cluster/cluster8/Enrich/ego_CC.pdf]

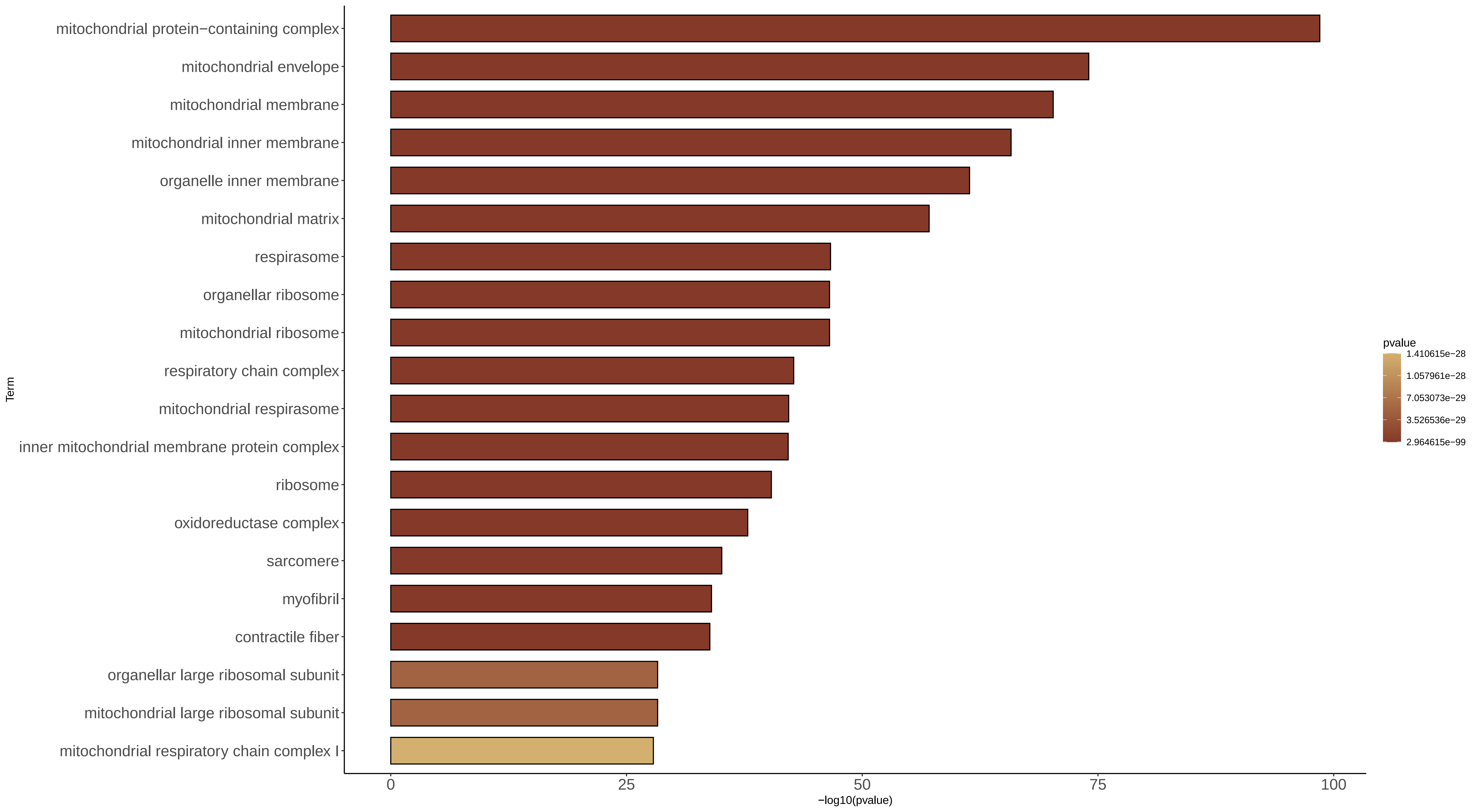

mitochondrial protein−containing complex
mitochondrial envelope
mitochondrial membrane
mitochondrial inner membrane
organelle inner membrane
mitochondrial matrix
respirasome
organellar ribosome
mitochondrial ribosome
respiratory chain complex
mitochondrial respirasome
inner mitochondrial membrane protein complex
ribosome
oxidoreductase complex
sarcomere
myofibril
contractile fiber
organellar large ribosomal subunit
mitochondrial large ribosomal subunit
mitochondrial respiratory chain complex I
Term
0
25
50
75
100
−log10(pvalue)
pvalue
1.410615e−28
1.057961e−28
7.053073e−29
3.526536e−29
2.964615e−99
